# Autistic feature as a presentation of Inborn Errors of Metabolism

**Published:** 2020

**Authors:** Farzad AHMADABADI, Hamid NEMATI, Amirmohammad ABDOLMOHAMMADZADEH, Adel AHADI

**Affiliations:** 1 Department of Pediatrics,Faculty of Medicine,Ardebil University of Medical Sciences, Ardebil, Iran; 2 Shiraz Neuroscience Research Center,Shiraz University of Medical Sciences, Shiraz, Iran; 3 School of Medicine, Iran University of Medical Sciences,Tehran, Iran; 4 Department of Pediatrics,Faculty of Medicine,Ardebil University of Medical Sciences, Ardebil, Iran

**Keywords:** Autism, Autism Spectrum Disorders, Inborn Errors of Metabolism

## Abstract

Autism spectrum disorder (ASD) is a category of neurodevelopmental disorders characterized by social and communication impairment and restricted or repetitive behaviors. The pathogenesis of ASD is not

well understood and it’s proved that genetic is strongly associated with ASD in 5 to 25% of cases.

Inborn errors of metabolism(IEMs), defined by a vast array of disorders that are caused by specific enzyme deficiencies or transport protein defects, is as frequent as in 1 in 800 births. IEMs can manifest

several psychiatric or behavioral manifestations such as self-injuriesincreased activity and aggression, personality changes, paranoia, depression, catatonia, and psychosis. IEMs underlie autistic symptoms in less than 5% of cases. The literature on the association between ASD and respiratory chain

abnormalities is growing, including complex III/IV deficiency and MELAS (mitochondrial encephalopathy, lactic acidosis, and stroke-like episodes) syndrome, as well as glucose-6-phosphate

dehydrogenase deficiency. Google Scholar, Pubmed, and SCOPUS databases were searched using a combination of the following keywords: “autism spectrum disorder”, “autism spectrum”, “autistic feature” and “inborn error of metabolism”, “ IEM”, “congenital error of metabolism”. Initially,

655 articles were found and our expert and methodologist altogether selected 187 articles based on the titles, relevance, and text language. After reading full texts, 37 studies were selected for review.

We think it’s best to consider IEMs in children with syndromic ASD and/or if there is a strong familial history of autism or parental consanguineous marriage.

## Introduction

Autism spectrum disorder (ASD) is a category of neurodevelopmental disorders characterized by social and communication impairments and restricted or repetitive behaviors.[1] Its prevalence in the US population is about 5 million, which is similar to other industrialized countries. Meanwhile, its prevalence is nearly 1.7% among children.[1]  The terminology and diagnostic criteria for ASD vary geographically, where The Diagnostic and Statistical Manual of Mental Disorders (DSM) is used predominantly in the United States (DSM-5, updated 2013)[2] and World Health Organization International Classification of Diseases, 10th revision (ICD-10) is used in other countries.[3] The DSM-5 category for identifying ASD is as follow [2]:According to the first criterion, the deficits must be clinically significant and persistent and should include the following: (1) marked impairment of both verbal and nonverbal communication so that social interaction be intervened; (2) lack of social reciprocity; and (3) failure to develop appropriate peer relationships. The second criterion consists of restrictive repetitive interests presented by at least 2 of the following: (1) stereotyped motor or verbal behaviors or unusual sensory behaviors, (2) excessive adherence to routines and ritualized patterns of behavior, and (3) restricted, fixated interests. Based on the third criterion, the symptoms of autism must be presented in early childhood. ASD is more common in boys than girls (a male/female ratio of 4.2)[4] and happens in nearly 10% of an ASD patient’s siblings.[5]

The pathogenesis of ASD is not well studied and a strong genetic caustic association is found at 5 to 25 percent of cases.[6] However; single-gene mutations only appear in approximately 5% of cases which suggests autism is a complex multifactorial disorder with multiple genetic factors involved as well as environmental factors that affect its etiology [7]. These factors include diagenetic (such as neuroanatomical and neuroimaging abnormalities), immunological, and metabolic factors.[8] Associated disorders include Fragile X syndrome, tuberous sclerosis, Angelman syndrome, duplication of 15q11-q13, Rett syndrome, Down syndrome, and Cohen syndrome.[9] Twin studies have reported that concordance for autism in monozygotic twins is 36 to 90%, which further highlights the influence of genetic-related factors.[10] However, it was assumed that environmental factors do not have an important role in findings such as the high prevalence of autism in monozygotic twins, which is partly due to the limited understanding of gene-environment interactions.[11]

To increase the diagnostic yield of autism, few issues are to be noted: 

First, a significant number of diagnoses can be made by reviewing the patient’s history and dysmorphology examination alone. Second, careful consideration of medical and genetic tests significantly increases diagnostic yield.

 Third, diagnostic yield increases with the aggressiveness of the evaluation.[6]

Inborn errors of metabolism(IEMs), defined by a vast array of disorders that are caused by specific enzyme deficiencies or transport protein defects, can be as frequently reported as in 1 in 800 births.[12] These conditions include organic acidemias, aminoacidopathies, urea cycle defects, disorders of carbohydrate metabolism, lysosomal storage disorders, beta-oxidation defects, peroxisomal disorders, and mitochondrial disorders. [13] Purine metabolism errors, such as adenylosuccinase deficiency and adenosine deaminase deficiency and congenital disorders of glycosylation, are other IEMs.[14]

IEMs can manifest several psychiatric or behavioral manifestations such as self-injuries- increased activity and aggression, personality changes, paranoia, depression, catatonia, and psychosis.[15]

IEMs cause the autistic features in less than 5% of cases. The relationship between ASDs and respiratory chain abnormalities, including complex III/IV deficiency and MELAS(Mitochondrial encephalopathy,Lactic acidosisand Stroke like episodes) are found as well as glucose -6-phosphate dehydrogenase deficiency in few cases.

Also, a number of reports suggest metabolic imbalances in ASD patients that indicate IEM, such as abnormalities of glucose oxidation and utilization. In other cases, it’s hard to distinguish IEMs as a primary or secondary pathology .[16] 

A study on 274 non-syndromic ASD children reported metabolic disturbances in only 2 children,[10] and the authors suggested to exclude routine metabolic screening for these patients. In another cohort of 32 patients who were referred for genetic evaluation with a diagnosis of autism, no patients had abnormal metabolic screening (including Urine organic acids and mucopolysaccharides, Serum lactic acid, amino acids, ammonia, and acyl-carnitine profile)[6].

Not so far ago, a unique set of biomarkers was found in ASD children with a proteomic approach that carried the potential for the early detection of ASD, even with the limited population of the study group.[17] Which then also might carry the potential for understanding the etiology or etiologies of ASD furthermore.

 The current study aimed to narratively review the associated metabolic conditions in ASD children with or without any previous condition.

**Methodology**

Google Scholar, PubMed, and SCOPUS databases were searched using the combinations of following keywords: “autism spectrum disorder”, “autism spectrum”, “autistic feature”, “inborn error of metabolism”, “ IEM”, and “congenital error of metabolism”. Initially, 655 articles were found and our expert and methodologist altogether selected 187 articles based on the correlation of titles and text language. After reading the abstract and full texts, a total of 49 studies were selected for review. The inclusion and exclusion criteria are shown in Table 1.

**Table1:** Inclusion& Exclusion criterias in our study

**Inclusion criteria**	**Exclusion criteria**
Articles that were about IEMs of metabolism, autism, and other matters of interest to the authors	Any language other than English
Availability of full text or abstract	Articles before 2009/ If the chronological view isn’t reviewed

**-Associated disorders**

We classified metabolic disorders that manifested autistic features in 10 groups (table 2).

**Table 2:** Classification of IEMs those presented with Autistic features

Aminoacidopathies	Organic Acidemia&Aciduria
Fatty acid Metabolism	Vitamins & Minerals Related Disorders
Carnitine related disorders	Mucopolysacharidosis
Mitochondrial Disorders	Urea Cycle defect
Creatin Related Disorders	Miscellaneous
Purine & Pyrimidine Disorders	

**Amino acid metabolism**

Autistic behavior has been in imbalances in neurotransmitter amines function such as dopamine, noradrenaline, and serotonin. Hyperserotonemia in particular shows a familial pattern and was found in over 25% of children and adolescents with autism. Amino acids can act as neurotransmitters in the central nervous system(CNS). In particular, glutamate, which can have an excitatory effect at high concentrations.[18, 19]

A study by Aldred, S. et al. analyzed amino acid levels of 12 autistic patients and 11 patients diagnosed with Asperger syndrome and their parents (n=32) and siblings(n=13). The authors reported that glutamic acid, phenylalanine, lysine, and asparagine were significantly higher (*p-value* 0.05) in both children and their parents compared to age-matched controls. Besides, they had lower levels of glutamine. The study also mentioned that the disrupted glutamate/glutamine ratio could be a predisposing factor in the etiology of autism. Glutamine is essential for enteric nutrition and many gastrointestinal problems are frequently reported in children with autistic features.[18]

- **Phenylalanine**

Phenylketonuria(PKU) is an autosomal recessive and the most common genetic metabolic disease which is associated with ASD.[20] Mental retardation, microcephaly, tremor, motor retardation, and ADHD are also reported to be associated with PKU. The PKU causes by dysfunction of phenylalanine hydroxylase (PAH), which is an essential enzyme in converting phenylalanine (PHE) to tyrosine. It increases the levels of PHE that can be harmful to brain development in first years of life, which causes variable neurologic manifestations and mental impairments. Imbalanced Excitation/inhibition(E/I) pathway is proposed as a potential pathophysiologic mechanism involved in ASD. A study that used a PKU model of mice (ENU2) further examined this mechanism by examining functional and molecular alterations in their prefrontal cortex and reported an imbalance in the E/I neurotransmission favoring inhibition in the prefrontal cortex of ENU2 mice and alterations of the molecular components involved in the organization of cortical synapse, which helped to clarify the pathology of ASD at least in animal models.[20] White matter lesions are also one of the suggested pathologies for behavioral and cognitive problems in PKU, which are further corrected by mentioning characteristics of the interaction of environmental, genetic, metabolic, neurological, and developmental factors in which vary in various cases. [21] Early correction of the PHE levels appears to cause improved behavioral and cognitive function in PKU, even if the PKU is diagnosed as late as the age of 3.[21]

- **Methionine, Cysteine, and Homocysteine**

The methionine-folate pathway defect have been identified in individuals with ASD. As a pathway that plays a critical role in DNA synthesis, methylation, and cellular redox balance, folate deficiency has been known as a plausible predictor for neurodegenerative diseases, such as Parkinson’s disease and Alzheimer’s.[11, 22] Methionine is an essential amino acid that is later converted S-adenosyl-methionine(SAM) and acts as the body’s main methyl group donor. During the methylation reactions, it converts to S-adenosyl-homocysteine(SAH). Homocysteine can later be converted to cysteine(and later to form glutathione) or remethylated to form methionine by methionine synthase(MS). Vitamin B12 is also necessary for this conversion.[22-24] An in-depth systematic review showed that defects in this cycle showed significantly lower levels of methionine, SAM and homocysteine and significantly higher levels of SAH in children with ASD compared to the controls and a later study (however in a lower population) showed no significant differences. As for the folate metabolism, treatment with folinic acid and betaine improves the concentration levels of metabolites from the methionine pathway in cases with impairments (some studies) and adding vitamin B12 further improved their levels. Significant behavioral improvements, however, were not observed. 

Vitamin B6, which is required for the conversion of THF ((tetrahydrofolate) to 5,10-MTHF( 5,10-methylene tetrahydrofolate formed from methylated THF and later mostly converted to 5-MTHF) and homocysteine to cysteine is significantly higher in some children with ASD compared to controls, which can indicate impairments. It is also important because impaired bioavailability of B6 can affect the CNS, because it is required for the synthesis of neurotransmitters such as serotonin, dopamine, and taurine.[22, 24, 25] In a case-control study, Omani has compared the levels of folate, homocysteine, and vitamin B12 in 40 ASD children and 40 matched controls and reported significantly higher levels of homocysteine, folate, and vitamin B12 in ASD children compared to the controls. Although nutritional factors may be important, defects in the folate-methionine cycle should also be considered.[23]

**Organic Acidemia**

- Propionic academia is one of the most common IEMs that may coexist with autism. In one study autism spectrum disorder was frequent in patients with propionic academia without any correlation between symptoms and 3-hydroxy propionate level.[26]
- Partial Biotinidase deficiencies were reported in a 4 year old girl with autistic feature. Severe forms of disease are life threatening, if not treated, and can result in many other symptoms.[27]
- L-2-Hydroxyglutaric aciduria (L-2-HGA) is a rare autosomal recessive metabolic disorder that affects exclusively the central nervous system and is characterized by a defect in the metabolism of L-2-hydroxyglutaric acid and, consequently, increased levels of the acid in the urine, cerebrospinal fluid, and plasma. Mild psychomotor delay in the first years of life, followed by progressive cerebellar ataxia, dysarthria, and moderate to severe mental deterioration, and macrocephaly, pyramidal and extrapyramidal signs, seizures, and dystonia are common in most of the patients. Language deficits, hyperactivity, and a talkative child are among the reported behavioral and communicative problems. A case of L-2-HGA has been reported who had manifested severe autism. The author reported that even though this finding could have been by chance, it is worthwhile for the physicians to note autism in children with L-2-HGA.[28]

**Mitochondrial & Fatty acid metabolism disorders**

The mitochondria is an intracellular organelle that plays a crucial role in adenosine 5′-triphosphate (ATP) production through oxidative phosphorylation (OXPHOS), which is carried out by electron transport chain(ETC).

IEMs contain two types of onsets: first is shortly after birth and second is when the child has a genetic background, but decompensation occurs later in the first years of life. Decompensation also can occur secondary to a serious illness or other major stresses. The second pattern can be consistent in the autistic regression seen in children with mitochondrial disease.[29] Mitochondrial disorders are also increasingly being recognized as a cause or related to the development and persistence of epilepsy, which is a common concomitant of ASD.[30]

A study that has reviewed the prevalence of mitochondrial disorders and ASD, theorized that if there was no association between ASD and mitochondrial-related diseases, it would be expected that 1 in 110 subjects with the mitochondrial disease would have been ASD and 1 in 2000 individuals with ASD would have mitochondrial disease. However, their results indicated a significant difference which suggested a possible shared pathology.[8]

Fatty acid beta-oxidation disorders are a group of inherited diseases that may either be caused by the failure of a single mitochondrial, peroxisomal enzyme of beta-oxidation or be secondary to dysfunction of dependent processes [31].

Mitochondrial fatty acid oxidation (FAO) deficiencies usually occur in neonates and toddlers and can cause psychomotor delay, developmental regression, behavioral disorders, and attention deficit as well as cardiomyopathy and muscle weakness. Long-chain acyl-CoA dehydrogenase (LACD) deficiencies are theorized in a case report, presenting an autistic patient with the abnormality of the acyl-carnitine profile, abnormal ammonia detoxification, and altered mitochondrial energy production together with hypotonia and possible intermittent dicarboxylic aciduria. The acyl-carnitine profile in this 8-year-old boy showed an abnormality that was mainly of an unsaturated specie, particularly C14:2 and C14:1. Although elevated C 14:1 could be seen in VLACD deficiencies, no other FAO deficiencies showed C 14:2. This finding led them to theorize that LACD deficiency can be the main cause, while cardiomyopathy and muscle weakness are prominent in other FAO-related disorders, such as VLCAD, increased physiologic turnover of unsaturated fatty acids in CNS, Gastrointestinal tract, and immune system which occur in autism, are more likely to justify these findings.[31, 32] Lower plasma levels of polyunsaturated fatty acids, elevations in polyunsaturated long-chain fatty acids, saturated very-long-chain fatty acids, and lipofuscin deposits in the prefrontal cortex have also been reported in ASD children compared to age-matched controls.[32]

- **Brain carnitine deficiency **

The main role of carnitine is to enable the transfer of fatty acids into mitochondria. Carnitine facilitates the entry of fatty acids into mitochondria by forming covalent acyl-carnitines. A study hypothesized that carnitine deficiency in the mitochondria of the brain, disturbs neurodevelopment, and cause Non-Mendelian, non-dysmorphic (NoMeND) autism, because previously low levels of carnitine are observed in ASD children and ASD was prevalent as common as in 3% of the cases of TMLHE deficiency (preventing the synthesis of carnitine from trimethyllysine). The results on animal models were favorable and the study suggested that early treatment with carnitine and other micronutrient supplementation may benefit recently symptomatic children and reduce the recurrence risk both in families and in the general population.[33]

- **Creatine transporter deficiency (CTD) and cerebral creatine deficiency syndromes (CDS)**

CTD is a known X-linked IEM that causes by mutations in creatine transporter gene (SLC8A8). It can cause disturbance of creatine import into the cells and cause problems in organs that require large amounts of energy such as the brain. Intellectual disability (ID), epilepsy, and autistic behavior and language disability are its main clinical presentations [34]. Increased creatine/creatinine ratio and normal urine guanidinoacetate levels are required for the diagnosis of CTD. on the other hand, one of the diagnosis criteria of CDS is decreased serum creatine levels, which is normal in patients with CTD. Clinical presentations of CDS include abnormal movements, developmental delays, seizures, and autism.[34]

Diagnosis is based on urinary and plasma guanidinoacetate and creatin levels as well as neuroimaging studies (MRS).

**Vitamins &Minerals**

Disorders in fetal brain development and lack of essential nutrition can later cause disorders such as neural tube defects and schizophrenia. Therefore, probably maternal nutritional imbalance affects the risk of ASD.[11] Whether maternal folate supplementation reduces the risk of ASD is still an open question with many factors that are hard to be singled out, however; there are studies which have suggested that folate supplementation can reduce the probability of ASD in children.[11, 25]

A study on 47 ASD children that were randomly assigned to treated with methycobalamin (methyl B12) at a dosage of 75 microgram/kg and saline placebo group every 3 days for 8 weeks reported significant improvements of CGI-improvement scale which was positively correlated with increased plasma methionine, decreased SAH, and improved SAM to SAH ratio [35]. 

**Muccopolysachariosis**

- Learning disorders and mental retardation are common presentations of Mucopolysaccharidosis (Hurler and Hunter syndromes);

- Sanfilippo syndrome or Mucopolysaccharidosis (MPS) type III is an inherited lysosomal storage defect that can cause severe neurodegenerative course. Patients with MPS type III have partially-degraded accumulated heparin sulfate fragments. MPS-IIIA and -IIIB patients of similar ages show similar neurocognitive impairment, and autistic features present in one-third of the children diagnosed with MPS type IIIA.[36]

**Smith–Lemli–Opitz syndrome (SLOS)**

Smith–Lemli–Opitz syndrome (SLOS) is an autosomal recessive condition caused by a defect in cholesterol synthesis leading to increased serum levels of 7-dehydrocholestrol. In a study, using the DSM-IV checklist, 14 children diagnosed with SLOS were evaluated for ASD and 7 of them scored at or above the cutoff for autistic disorder. They suggested that the observed association between autism and a defect in cholesterol metabolism suggests that cholesterol metabolism is not only linked to the pathogenesis of autism in SLOS, but also to autism in general.[9]

**Androgen theory of autism**

The androgen theory of autism proposes that autism spectrum conditions (ASC) are in part due to elevated fetal testosterone (FT) levels. Significantly higher male to female ratio sparked the idea that the autistic brain might be an “extreme” of the typical male brain. Increased FT measured in amniotic fluid can highly be correlated with the amount of eye contact at 12 months of age, vocabulary size at 18 and 24 months, and quality of social relationships at 4 years. It also correlates with some autistic features[37]. A survey on 54 women with ASC, 74 mothers of ASC children, and 183 mothers of normal children which used Testosterone-related Medical Questionnaire (TMQ) showed significantly higher rates of hirsutism, polycystic ovarian syndrome, and other androgen-related conditions in most cases and conditions. It also argued that elevated testosterone level is a common risk factor for hyperandrogenism in adult women and those with autism, rather than any of the conditions such as hirsutism causing autism.[37] 

**Table 3:** Clues for metabolic based autistic features

**Coexist symptoms**	**Lab Tests**	**Diagnosis**
ASD+Microcephaly	HPLC of AA	PKU,Serin deficiency,
ASD +Abnormal Movements+Epilepsy	Creatin Pannel	Cerebral creatin Deficiency syndromes
Distinct Syndromic appearence	Cholestrol pannel	Smith lemli opitz
Self Mutilation	Uric acid	Lesh nyhan syndrome
Coarse Facies +ASD	Urin Mucopolysacharides	Sanfilippo
ASD+acidosis	Biotine panel Urinary organic acids Lactat	Biotinidase deficienct Organic academia Mitochondrial disorders Lactate pathway
Macrocytic anemia+ASD	B12,Folate	MMA Homocystinuria Vitamins deficiency

**Figure 1:**
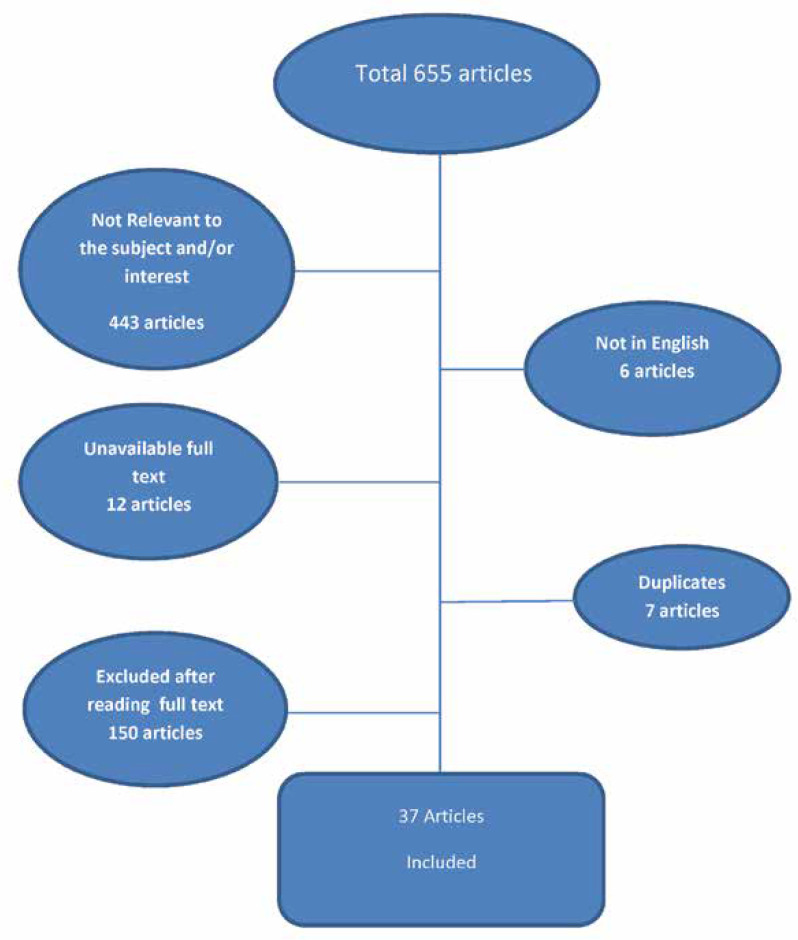
Algorithm of narrative review search about participation of articles

## Conclusion

In conclusion, although IEM may be relate to a small number of ASDs in general,; we can’t ignore the growing evidence about IEMs association with ASD. We think it’s best to consider IEMs in children with syndromic ASD and/or if a strong familial history of autism is observed, consanguineous marriage, and clinical suspicion. Besides, performing standard metabolic screening would be useful in these cases.(Table 3)
